# Artificial intelligence-enhanced electrocardiogram for arrhythmogenic right ventricular cardiomyopathy detection

**DOI:** 10.1093/ehjdh/ztad078

**Published:** 2023-12-09

**Authors:** Ikram U Haq, Kan Liu, John R Giudicessi, Konstantinos C Siontis, Samuel J Asirvatham, Zachi I Attia, Michael J Ackerman, Paul A Friedman, Ammar M Killu

**Affiliations:** Department of Cardiovascular Medicine, Mayo Clinic, 200 First Street SW, Rochester, MN 55905, USA; Department of Cardiovascular Medicine, Mayo Clinic, 200 First Street SW, Rochester, MN 55905, USA; Department of Cardiovascular Medicine, Mayo Clinic, 200 First Street SW, Rochester, MN 55905, USA; Department of Cardiovascular Medicine, Mayo Clinic, 200 First Street SW, Rochester, MN 55905, USA; Department of Cardiovascular Medicine, Mayo Clinic, 200 First Street SW, Rochester, MN 55905, USA; Department of Cardiovascular Medicine, Mayo Clinic, 200 First Street SW, Rochester, MN 55905, USA; Department of Cardiovascular Medicine, Mayo Clinic, 200 First Street SW, Rochester, MN 55905, USA; Department of Cardiovascular Medicine, Mayo Clinic, 200 First Street SW, Rochester, MN 55905, USA; Department of Cardiovascular Medicine, Mayo Clinic, 200 First Street SW, Rochester, MN 55905, USA

**Keywords:** AI-ECG, Arrhythmogenic right ventricular cardiomyopathy

## Abstract

**Aims:**

ECG abnormalities are often the first signs of arrhythmogenic right ventricular cardiomyopathy (ARVC) and we hypothesized that an artificial intelligence (AI)-enhanced ECG could help identify patients with ARVC and serve as a valuable disease-detection tool.

**Methods and results:**

We created a convolutional neural network to detect ARVC using a 12-lead ECG. All patients with ARVC who met the 2010 task force criteria and had disease-causative genetic variants were included. All case ECGs were randomly assigned in an 8:1:1 ratio into training, validation, and testing groups. The case ECGs were age- and sex-matched with control ECGs at our institution in a 1:100 ratio. Seventy-seven patients (51% male; mean age 47.2 ± 19.9), including 56 patients with PKP2, 7 with DSG2, 6 with DSC2, 6 with DSP, and 2 with JUP were included. The model was trained using 61 case ECGs and 5009 control ECGs; validated with 7 case ECGs and 678 control ECGs and tested in 22 case ECGs and 1256 control ECGs. The sensitivity, specificity, positive and negative predictive values of the model were 77.3, 62.9, 3.32, and 99.4%, respectively. The area under the curve for rhythm ECG and median beat ECG was 0.75 and 0.76, respectively.

**Conclusion:**

Our study found that the model performed well in excluding ARVC and supports the concept that the AI ECG can serve as a biomarker for ARVC if a larger cohort were available for network training. A multicentre study including patients with ARVC from other centres would be the next step in refining, testing, and validating this algorithm.

## Short report

Arrhythmogenic right ventricular cardiomyopathy (ARVC) is an inherited cardiomyopathy characterized by fibrofatty myocardial replacement, leading to progressive ventricular dysfunction and potentially lethal scar-mediated ventricular arrhythmias.^1^ Amongst young and athletic patients, ARVC represents one of the most common causes of sudden cardiac death.^1^ However, accurate diagnosis is challenging because of the highly variable clinical presentation, low prevalence, overlap with other cardiomyopathies, and lack of a single diagnostic test. The International Task Force criteria were created in 1994 and modified in 2010 to facilitate ARVC diagnosis, including electrocardiographic (ECG), imaging, histological, genetic, and familial features.^2^ Subtle depolarization and repolarization ECG abnormalities are often the first signs of ARVC and precede other task force criteria.^3^ Suggestive ECG features include T wave inversion in right precordial leads (V1–V3) in the absence of right bundle branch block, epsilon waves, localized prolongation (>110 ms) of QRS in right precordial leads, paroxysmal episodes of ventricular tachycardia with left bundle branch block morphology, and ventricular ectopy more than 1000 per 24 h.^3^ We hypothesized that an artificial intelligence (AI)-enhanced ECG could help identify patients with ARVC and serve as a valuable disease-detection tool.

To test this hypothesis, we trained, internally validated, and tested a convolutional neural network (CNN) to detect ARVC using the 12-lead ECG. The CNN was built using a total of eight stacked blocks of 2D convolutional layers, max pooling, batch normalization, and dropout layers. This was followed by two fully connected layers and a final output layer activated using a Softmax function to generate two outputs (negative or positive). We used the categorical cross entropy as the loss function. Batch size was chosen at 16 based on the cohort size. Learning rate was assigned as 3e^−4^ with Adam optimizer and weight decay regularizer. To better fit the model’s shape manipulation, all ECGs were zero-padded from 5000 × 12 × 1 to 5120 × 12 × 1 to maintain an even dimension after each pooling layer. A dropout rate was used at 0.5 and 30 epochs for training the network, while saving the model weights for the epoch that achieved the highest area under the curve [area under the curve of the receiver operating characteristic (AUC ROC)] on the internal validation set. Rhythm ECG and a median beat created by sample-by-sample medians of all P-QRS-T wave forms of all normal, noise-free beats recorded were studied separately. The median beats that were generated by MUSE (GE Healthcare) were visually reviewed to ensure adequate detection of individual P-QRS-T wave forms. The input was a matrix of amplitudes for each lead; each matrix contained the 10 s sampled at 500 Hz (5000 samples) for each lead (for all 12 leads) creating a matrix of [5000 × 12]. For the median beat model, we used 1.2 s (600 samples) centred at the R wave, for each lead of the 12-lead median beats—creating a matrix of [600 × 12]. All amplitudes were saved in μV.

**Figure 1:**
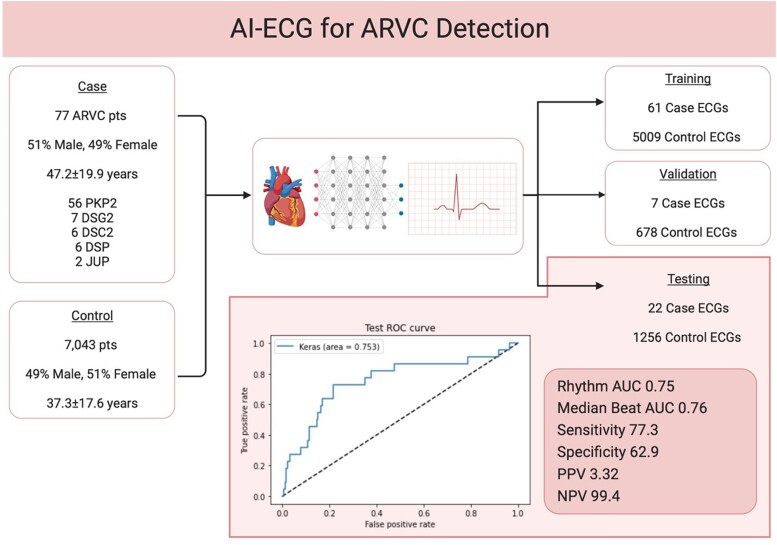
AI-ECG for Arrhythmogenic right ventricular cardiomyopathy detection. AI, artificial intelligence; ARVC, arrhythmogenic right ventricular cardiomyopathy; AUC, area under the curve.

We included all patients diagnosed with ARVC who met the 2010 task force criteria and had disease-causative genetic variants in definitive/strong evidence desmosomal genes with at least one digital, standard 10 second 12-lead ECG performed at our institution. Many patients underwent multiple ECGs and all ECGs were used in training the model but only the first ECG per patient was utilized during internal validation and testing. All study ECGs were randomly assigned in an 8:1:1 ratio into training, internal validation, and testing groups at the patient level, and no patient was used in more than one dataset. The study ECGs were age- and sex-matched with all other available ECGs at our institution in a 1:100 ratio. Based on the control poll ECG size limit, in some cases, the ratio was lower than expected. The primary outcome of the study was the ability of the AI algorithm to identify patients with ARVC using a standard ECG. The performance was mathematically assessed using the AUC ROC curve. The sensitivity, specificity, and positive and negative predictive values were also calculated (*Figure 1*).

The study cohort included 77 patients (51% male, 49% female; mean age 47.2 ± 19.9). All patients had an ARVC-causative genetic variant(s), including 56 patients with plakophilin-2 (PKP2), 7 with desmoglein-2 (DSG2), 6 with desmocollin-2 (DSC2), 6 with desmoplakin (DSP), and 2 with plakoglobin (JUP). The CNN model was trained using 61 ECGs from 48 cases and 5009 control ECGs. The model was subsequently validated in 7 ECGs from 7 cases and 678 ECG controls and then tested in 22 ECGs from 22 cases and 1256 ECG controls. The algorithm was run twice, first using all ECG leads and then repeated using only truly algebraically independent ECG leads. When all ECG leads were used, the sensitivity, specificity, and positive and negative predictive values were 77.3, 62.9, 3.32, and 99.4%, respectively. The AUC for rhythm ECG and median beat ECG was 0.75 and 0.76, respectively. When only algebraically independent ECG leads were used, the sensitivity, specificity, and positive and negative predictive values were 68.8, 60.6, 16.9, and 94.3%, respectively. The AUC for rhythm ECG and median beat ECG was 0.53 and 0.65, respectively.

Our study found that the AI-enhanced ECG performed well in excluding ARVC. A real-world study of the 2010 task force criteria found that the ECG criteria had a very high sensitivity for ARVC diagnosis, whereas echocardiography and cardiac magnetic resonance imaging had a higher specificity.^3^ However, ECG changes attributed to ARVC early in the disease course are subtle and difficult to discern from other repolarization/depolarization abnormalities. If a larger cohort were available and the specificity of the AI-enhanced ECG improved, the algorithm could potentially serve as an initial rule-out tool in identifying appropriate patients for a more focused diagnostic evaluation using other task force criteria to ‘rule in’ ARVC. This stepwise evaluation has the potential to detect for ARVC earlier in its disease course, reduce false-positive diagnosis driven by overinterpretation of cardiac imaging, and save time and resources, most notably in reducing the need for serial cardiac imaging.

The principal limitation of our study pertains to the low disease prevalence of ARVC, which was reflected in our relatively small study cohort, and the small number of affected ECGs, which impaired the learning capability of the neural network and the biases incorporated with a small study population. Nonetheless, these observations support the concept that the AI ECG can serve as a biomarker for ARVC if a larger cohort were available for network training. A multicentre study including patients with ARVC from other centres would be the logical next step in further refining, testing, and validating this algorithm.

**Conflict of interest:** No conflicts of interests related to this algotihm. However, KCS, SJA, ZIA and PAF are co-inventors on AI-ECG algorithms via Mayo Clinic and could benefit from their commercialization.

## Data Availability

The data that supports the findings of this study are available from the corresponding author upon reasonable request.
